# The effectiveness of vitamin C in dental alveolus healing after dental extraction: A scoping review

**DOI:** 10.4317/medoral.26893

**Published:** 2024-12-24

**Authors:** Jessika Dethlefs-Canto, Fernanda Osses-Barría, Rodrigo Vergara-Zenteno, Alexis Bustos-Ponce, Javier Villavicencio-Duarte

**Affiliations:** 1 Department of Oral and Maxillofacial Surgery, University of Valparaíso, Valparaíso, Chile; 2 Dentistry Student, Dentistry Faculty, University of Valparaíso, Valparaíso, Chile; 3 Hospital de Urgencia Asistencia Pública, Santiago, Chile; 4 Hospital San Camilo, San Felipe, Chile

## Abstract

**Background:**

Dental extraction is a common procedure in dentistry. It is accompanied by postoperative pain and inflammation. In addition, it decreases bone volume and density. Vitamin C is an antioxidant and cofactor that promotes the synthesis and maturation of collagen, the proliferation and migration of fibroblasts and osteoblasts, accelerating the final phase of inflammation, promoting healing. The objective of this scoping review is to evaluate the effectiveness of vitamin c in dental alveolus healing after extraction and synthesize the available evidence and tits clinical implications.

**Material and Methods:**

This review is registered on the Open Science Framework platform (https://osf.io/bstwk/). It was carried out under the PRISMA-ScR protocol, using the question: Is vitamin C effective in alveolar healing in patients undergoing dental extraction? The Pubmed/MEDLINE, Scopus, Web of Science and OPENGREY databases were used. Limiting itself to primary studies.

**Results:**

A total of 287 articles were identified, applying selection criteria, 3 were included. Of a total of 135 patients, 59.8% were administered vitamin C; 46.6% 600 mg, 34.2% 500 mg and 19.2% 1500 mg. A decrease in pain, inflammation, probing depth and mesiodistal length of the socket was observed.

**Conclusions:**

The role of vitamin C is essential for healing, and therefore, bone regeneration after tooth extraction, reducing adverse effects such as pain and inflammation. Its administration is recommended to promote postoperative recovery. More studies are suggested to observe its effects in oral and maxillofacial surgery.

** Key words:**Ascorbic acid, tooth extraction, wound healing, bone regeneration.

## Introduction

Alveolar healing after tooth extraction is a complex and essential biological process for patient recovery. This process involves a series of coordinated events, including blood clot formation, inflammation, cell proliferation, and tissue remodeling, all aimed at restoring the integrity of the affected tissue (1). However, post-extraction complications, such as dry socket, infections, and prolonged pain, can hinder healing and extend recovery time (1).

Ascorbic acid, better known as vitamin C, has been extensively studied due to its well-known antioxidant properties and its essential role in collagen synthesis. This essential micronutrient acts as a cofactor in enzymatic reactions necessary for the hydroxylation of proline and lysine, amino acids crucial for collagen stability and functionality (2). Additionally, vitamin C plays a protective role by neutralizing reactive oxygen species (ROS), which can damage cells and tissues, creating a favorable environment for healing (3).

Previous studies have shown that vitamin C supplementation can accelerate the healing of skin wounds and ulcers, improve immune response, and reduce inflammation (4). In studies on skin wounds, vitamin C has been shown to promote healing through novel pleiotropic mechanisms, including inflammation modulation and fibroblast proliferation promotion (5).

In the field of dentistry, its application has generated considerable interest, especially regarding its potential to enhance alveolar healing after tooth extraction. However, the results of these studies have been varied and, in some cases, contradictory. While some research reports significant improvements in healing and a reduction in postoperative complications with the use of vitamin C, others do not find statistically significant differences compared to controls (3). Likewise, studies on healing in the context of dental implants have indicated that vitamin C could play an important role in bone regeneration and implant stability (2). Additionally, *in vitro* studies have shown that rinsing with ascorbic acid exhibits concentration-dependent effects on the healing behavior of human gingival fibroblasts, suggesting a potential benefit for alveolar healing (6).

The use of vitamin C as a postoperative supplement in the context of dental extractions could represent a significant advancement in dental practice, offering an accessible and potentially effective therapeutic option for improving clinical outcomes. By synthesizing the available evidence, this review aims to clarify the utility of vitamin C in alveolar healing and provide evidence-based recommendations for its implementation in clinical practice (6).

## Material and Methods

The PCC question formulation was done as follows: Is vitamin C effective in alveolar healing in patients undergoing tooth extraction?

People: Patients undergoing tooth extraction

Concept: Vitamin C treatment

Context: Not applicable

**Figure 1:**
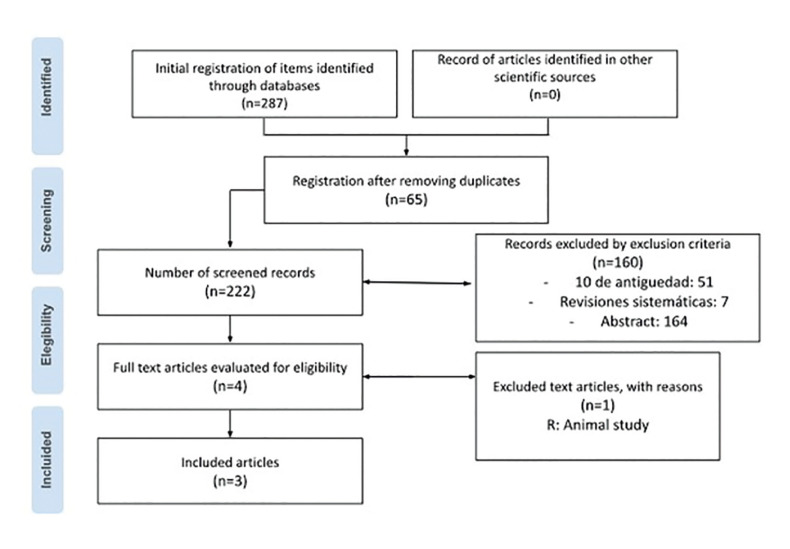
PRISMA Flowchart.

A scoping systematic review was conducted, using the elements for the development of reviews under the PRISMA-ScR protocols, registered on the Open Science Framework platform (https://osf.io/bstwk/) (Fig. 1).

The following MESH terms were used for the search: “Tooth Extraction,” “Ascorbic Acid,” and free terms like “Vitamin C,” “Bone Regeneration,” and “Alveolar Healing,” along with the Boolean connectors “AND” and “OR” in the following databases: Scopus, PUBMED/MEDLINE, Web of Science, and OPENGREY, with a maximum article age of 10 years, and the human studies filter applied.

The search yielded 287 results, which were included in the Rayyan virtual tool (https://new.rayyan.ai) to remove duplicate studies. Subsequently, a title and abstract review was conducted, selecting 222 studies. After reading the full texts of the selected articles, 7 studies were eliminated for being systematic reviews, 51 for age, and 160 for not being relevant to the research question, leaving 5 articles. One article was excluded because it was conducted on animals, ultimately leaving 4 articles for full reading, as shown in Fig. 1.

Primary studies, specifically randomized clinical trials (RCTs), conducted in English and Spanish were included. Book chapters, letters to the editor, and case reports were excluded.

The studies conducted on individuals who underwent dental extractions and were treated with vitamin C as part of the post-extraction medicinal therapy were included in this review. Additionally, studies describing clinical outcomes derived from vitamin C administration for post-extraction treatment were included.

The analyses described above were carried out by two reviewers individually and independently. In case of disagreement, the inclusion and exclusion were jointly analyzed according to the previously established criteria, as well as the relevance of the study and its correlation with the research question and objectives.

## Results

A study of thirty patients who underwent bilateral premolar extractions were divided into three groups: systemic vitamin C administration, a combination of local and systemic administration, and control (Table 1). The results showed that combined vitamin C administration significantly improved soft tissue healing, decreased probing depth of the alveolus, and increased radiographic bone density 21 days post-extraction compared to the control group (7).

Another study evaluated the effect of vitamin C (200 mg three times a day) on the healing of extraction sockets. The results showed significant improvements in wound healing parameters, a decrease in probing depth and mesiodistal length of the socket, including a reduction in inflammation and pain, and better bone tissue regeneration compared to the control group (8).

When evaluating the anti-inflammatory efficacy of ascorbic acid after impacted third molar surgery, the results indicated that vitamin C significantly reduced inflammation and postoperative pain. Additionally, a decrease in C-reactive protein values was observed in the intervened patients compared to the control group (9).

Finally, a study investigated the local application of propolis extract, nanovitamin C, and nanovitamin E to prevent alveolar osteitis after mandibular impacted third molar surgery. The results showed that nanovitamin C application was effective in reducing the incidence of alveolar osteitis and pain reduction (10).

## Discussion

The results of these studies underscore the importance of vitamin C as an effective therapeutic agent for improving wound healing after dental extractions and implants. The administration of vitamin C, both systemic and local, has significantly improved soft tissue healing and bone regeneration, which is crucial for clinical success (7,8,10,11)

The positive effect of vitamin C can be attributed to its role in collagen synthesis, reducing oxidative stress, and modulating the inflammatory response. These mechanisms are essential for tissue regeneration and wound healing, especially in settings where healing may be compromised, such as in patients with chronic periodontitis or those requiring bone grafts (2,3)

Furthermore, studies have indicated that combining local and systemic vitamin C administration may offer additional benefits compared to systemic administration alone, improving the absorption and effectiveness of vitamin C at the wound site (7). This suggests that combined administration strategies could be more effective in optimizing post-extraction healing.

Administering vitamin C three times a day significantly improves extraction socket healing parameters (8), consistent with other studies suggesting a positive effect of vitamin C on bone tissue regeneration and inflammation reduction.

The current evidence on the anti-inflammatory and analgesic benefits of vitamin C indicates that it may be a viable alternative to conventional treatments for managing postoperative pain (7,8,10,11)

Vitamin C supplementation represents a viable and effective intervention to improve wound healing after dental extractions and implants, offering significant benefits in terms of pain reduction, inflammation, and improvement in soft and bone tissue regeneration. Future research could focus on optimizing doses and administration methods to maximize these clinical benefits and address the limitations of these studies.

## Figures and Tables

**Table 1:** Results.

Author, Year	Language	Study design	Sample	Objective	Intervention	Results and conclusion
Fatima et al., 2023	English	Observational, cross-sectional study	50 patients. Healthy. Over 18 years. Impacted third molar extraction.	To assess the anti-inflammatory effect of ascorbic acid following surgical extraction of the third molar.	Comparison between vitamin C oral tablet 500 mg and control group.	Significant reduction in pain and inflammation at 7 days. No significant differences in C-reactive protein value.
Yingcharoenthana et al., 2021	English	Randomized, controlled clinical trial.	30 patients. Healthy. Mean age 20.07 ± 2.66. Healthy premolars extraction.	To investigate the efficacy of local and systemic vitamin C administration in the healing of tooth extraction wounds based on the remodeling of soft and bone tissues.	Comparison between systemic, local/systemic and control group absorption of vitamin C oral tablet 600 mg	Reduction of probing depth of the alveolus in operated patients. Changes in radiographic bone density in operated patients at 21 days, with no statistically significant differences. Local/systemic application results in better healing.
Pisalsitsakul et al., 2022	English	Randomized clinical trial.	42 patients. Healthy. Mean age 18.68 ± 3.95. Healthy premolars extraction.	To investigate the effect of different oral doses of vitamin C on post-extraction wound healing.	Comparison of vitamin C oral tablet 600 mg, 1500 mg and control group.	There is no significant difference between doses of 600 and 1500 mg. 600 mg group demonstrated a significant reduction in mesiodistal measurement of the alveolus and postoperative pain between day 0 and 7, versus placebo group.
González-Serrano et al., 2021	English	Randomized clinical trial.	13 patients. Healthy. Mean age 21.15 ± 2.03. Impacted mandibular third molars extraction	To evaluate the effectiveness and safety of the gel (2% propolis extract, 0.2% ascorbic acid and 0.2% tocopherol acetate) in the control of postoperative complications in patients undergoing extraction of mandibular third molars.	Comparison of gel application and control group.	Decrease in alveolar osteitis in operated patients, with no statistically significant difference. Significant decrease in pain in the operated group.
